# Delivery of cardiac rehabilitation through hybrid models and alternative settings by country income classification and decade: Comparisons from the International Council of Cardiovascular Prevention and Rehabilitation’s 2025 Global Audit Update

**DOI:** 10.1371/journal.pone.0359951

**Published:** 2026-10-09

**Authors:** Gabriela L. M. Ghisi, Rachael P. Carson, Karam Turk Adawi, Rongjing Ding, Monserrat Cruz Rivero, Claudia V. Anchique, Dinah L. van Schalkwijk, Jonathan Gallagher, Chamila Mettananda, Rosalia Fernández, Gulsim T. Aimakova, Won-Seok Kim, Pamela Seron, Mariya P. Jiandani, Jimena Martinez, Lela Maskhulia, Ladislav Batalik, Buket Akinci, Ssu-Yuan Chen, Ngone Diaba Gaye, Daniel F. Quesada-Chaves, Yoshihiro Fukumoto, Sherry L. Grace, on behalf of the ICCPR Global Cardiac Rehabilitation Audit Update Investigators

**Affiliations:** 1 The Jay and Sari Sonshine Centre for Stroke Prevention & Cerebrovascular Brain Health, Toronto Western Hospital, University Health Network, University of Toronto, Toronto, Canada; 2 Department of Physical Therapy, University of Toronto, Toronto, Canada; 3 Faculty of Health, York University, Toronto, Canada; 4 Department of Public Health, College of Health Science, QU Health, Qatar University, Doha, Qatar; 5 Cardiac Rehabilitation Center, Peking Union Medical College Hospital, Beijing, China; 6 Casa Corazón Otoch Puksikal, Mérida, Mexico; 7 Mediagnóstica Tecmedi SAS, Duitama, Colombia; 8 Department of Cardiology, Radboud University Medical Center, Nijmegen, The Netherlands; 9 Department of Medical and Clinical Psychology, Tilburg University, Tilburg, The Netherlands; 10 Department of Cardiology (Cardiac Rehabilitation), Beaumont Hospital, Dublin, Ireland; 11 Department of Pharmacology, Faculty of Medicine, University of Kelaniya, Ragama, Sri Lanka; 12 Cardiac Rehabilitation, National Cardiovascular Institute–INCOR, Lima, Perú; 13 San Fernando Medical School, National University of San Marcos, Lima, Perú; 14 UMC Heart Centre, Astana, Kazakhstan; 15 Department of Rehabilitation Medicine, Seoul National University College of Medicine, Seoul National University Bundang Hospital, Seongnam-si, South Korea; 16 Gyeonggi Regional Cardiocerebrovascular Disease Center, Seoul National University Bundang Hospital, Seongnam-si, South Korea; 17 Departamento de Ciencias de la Rehabilitación, Universidad de La Frontera, Temuco, Chile; 18 Department of Physical Therapy, Seth GS Medical College and KEM Hospital, Mumbai, India; 19 Instituto Medico DAMIC, Cordoba, Argentina; 20 Physical Medicine, Tbilisi State Medical University, Tbilisi, Georgia; 21 Department of Rehabilitation, University Hospital Brno, Brno, Czech Republic; 22 Rehabilitation Clinic, Faculty of Medicine, Masaryk University, Brno, Czech Republic; 23 Physiotherapy and Rehabilitation Department, Faculty of Health Sciences, Biruni University, Istanbul, Turkey; 24 Biruni University Research Center, Biruni University, Istanbul, Turkey; 25 Cardiovascular and Pulmonary Rehabilitation Center and Division of Physical Medicine and Rehabilitation, Fu Jen Catholic University Hospital, New Taipei City, Taiwan; 26 Department of Physical Medicine and Rehabilitation, National Taiwan University Hospital and National Taiwan University College of Medicine, Taipei, Taiwan; 27 Cardiac Rehabilitation, Ibra Mamadou Wane Medical Center, Dakar, Senegal; 28 Hospital San Vicente de Paul, Heredia, Costa Rica; 29 Division of Cardiovascular Medicine, Department of Internal Medicine, Kurume University School of Medicine, Kurume, Japan; 30 KITE - Toronto Rehabilitation Insitute and Peter Munk Cardiac Centre, University Health Network, University of Toronto, Toronto, Canada; University of Illinois Urbana-Champaign, UNITED STATES OF AMERICA

## Abstract

**Objective:**

Cardiac rehabilitation (CR) is an outpatient model of secondary prevention that improves patient outcomes, yet use remains limited. Hybrid/alternative CR addresses these challenges, but global availability, structure, and volumes have not been characterized since the pandemic.

**Methods:**

The International Council of Cardiovascular Prevention and Rehabilitation’s (ICCPR) 2025 Audit Update is a cross-sectional survey of phase II CR programs worldwide. After member Associations supported identification of countries with CR, program data were collected via REDCap between May-September/2025. Data were compared by country income classification, and with prior ICCPR Audits where possible.

**Results:**

Of 6,905 CR programs identified globally, 1,505 initiated a survey (median program response rate = 62.3% per country) from 90/113 (79.6%) countries with CR. Globally, 286 (23.2%) programs in 40 (35.4%) countries reported reimbursement for hybrid/alternative models, varying significantly by country income class (p < 0.001); this was higher than 2016 (14.5%), but not significantly different from the pandemic (21.0%). **285 (31.1%)** programs reported offering a hybrid/alternative CR model, in 57 (50.4% with CR) countries (higher in lower-middle income countries, then upper-middle income countries [UMICs]). This was not higher than previous Audits. Programs were staffed by exercise professionals, nurses and physicians (more in UMICs). Dose was a median of 10 sessions (one/week over 8 weeks), lower than centre-based, but consistent across income classes and over time. Patients were allocated to model based on clinical criteria, choice (69.5%), but also cost (30.2%; higher in middle-income countries and than 2016). Hybrid/alternative models served a median of 50 (P25-P75 = 10–100) patients/program annually, lower than centre-based (median = 135; p < 0.001); volumes were higher in UMICs. 160 (45.8%) programs perceived insufficient capacity to meet demand, most commonly due to staffing and funding.

**Conclusions:**

Hybrid/alternative CR availability has not increased since 2016, and decreased since the pandemic. Reimbursement drives availability, but remains limited and inadequate, frequently shifting costs to patients, constraining services and limiting equitable scale-up.

## Introduction

The increasing incidence and reduced case fatality of cardiovascular diseases (CVD) have resulted in a growing number of people globally living with CVD as a chronic condition [1]. Consequently, there is an increasing need for secondary prevention to optimize both quantity and quality of life among people living with CVD. The disease, health system, and economic burden of CVD is particularly high in low- and middle-income countries, which account for approximately 80% of cardiovascular deaths globally [2].

Cardiac rehabilitation (CR) is a multi-phase continuum of care spanning inpatient, early outpatient, and longer-term community-based secondary prevention services. Phase II represents the early outpatient stage of CR and provides a structured secondary prevention program, delivered over approximately two months on average globally [3], to support comprehensive risk-factor management and return to life roles [4]. Participation is associated with reduced cardiovascular mortality, hospital readmissions, and improve quality of life [5].

Traditionally, CR has been delivered in clinical settings, most commonly within a hospital. Globally, approximately 80% of programs are hospital-based [6]. Despite its proven benefits, CR remains markedly under-utilized [7]. Common barriers to participation include distance from CR centres, transportation challenges, and competing work or time commitments [8,9] – barriers that disproportionately affect patients in rural regions, those of lower socioeconomic status, and working-age populations.

To address these barriers, home-based models have increasingly been implemented. Home-based CR refers to a structured program in which core CR components are delivered outside of a traditional centre, with patients monitored and supported remotely by healthcare professionals [10,11]. Hybrid models combining centre- and home-based care have also become more commonly offered. These models may be delivered with or without digital technologies, such as synchronous supervised group exercise classes via video streaming. In addition, some programs offer services in community settings, which too can mitigate barriers related to distance and transportation costs. These models were designed chiefly to mitigate patient-level CR barriers to access [8,14]. Evidence shows that hybrid and/or alternative models of CR are safe [15], achieve clinical outcomes comparable to traditional centre-based models [12], and may be lower cost than traditional centre-based models [12,13].

The International Council of Cardiovascular Prevention and Rehabilitation (ICCPR) conducted a first-ever Global Audit of CR delivery in 2016 [16,17]. It included assessment of hybrid and/or alternative CR models. Programs in 51 countries with CR (45.9%; or 25.1% of all countries globally) offered any such models, with 31% of programs globally indicating their availability [18]. Since the COVID-19 pandemic, adoption of technology-enabled CR delivery increased substantially to reduce infectious risk. Indeed, during ICCPR’s COVID-19 Rapid Audit, approximately 40% of programs reported delivering hybrid and/or alternative models, primarily through low-technology approaches. However, the extent to which these models are currently implemented worldwide remains unknown, particularly given their historically limited reimbursement [18,19].

Therefore, this 2025 ICCPR Audit Update sub-study focused on the implementation of hybrid/alternative models globally. The objectives were to describe globally phase II CR hybrid/alternative model: (1) reimbursement, (2) availability, (3) nature / implementation of model services (i.e., structure, staffing, communication); (4) dose; (5) patient model allocation criteria; as well as (6) annual patient volumes and capacity. Findings were examined by: (a) country income classification, and where possible, compared with (b) center-based models and/or (c) the prior ICCPR Audits [16,17].

## Methods

### Design & procedure

This Global Audit Update was cross-sectional and observational in design. The protocol was approved by ICCPR Executive and publicly posted prior to study initiation (https://globalcardiacrehab.com/2025/26-Audit-Update). Ethical approval was obtained from York University (Toronto, Canada; e2025-057). Procedures are depicted elsewhere (“Turk-Adawi K, Ghisi GLM, Arar N, et al. [Unpublished]”). Audit findings regarding CR capacity and coverage (“Turk-Adawi K, Ghisi GLM, Arar N, et al. [Unpublished]”); referrals and access [6]; staffing, indications and dose [3]; as well as core components [20] are reported elsewhere (results by region are forthcoming [21]).

The World Health Organization’s list of countries [22] was used as a basis for the Audit (with United Kingdom countries counted separately herein; N = 199). To determine which countries had existing CR programs, those reported to have any program availability in previous Audits (111 of 203 countries) [16,17] were first considered. CR and related Societies (including ICCPR members), or other national leaders identified through peer-reviewed publications, ICCPR’s network including umbrella Associations (e.g., International Society of Physical Medicine and Rehabilitation), or organizational websites verified any national phase II CR service provision, and if affirmative the number of programs meeting Audit inclusion / exclusion criteria. In countries without CR, regional leads confirmed its current absence, or a local contact was made if disconfirmed.

Identified leaders in countries with ≤9 CR programs were asked to share the contact email for each program in return for free ICCPR CR Foundations Certification (CRFC; value $125USD) [23]. Identified leaders in countries with ≥10 CR programs were asked to ascertain and share the program email addresses or distribute the survey invitation letter and link with their Society program email distribution list. Those who contributed were invited to collaborate to ensure accurate and fulsome dissemination of the results. Societies were offered a personalized national comparative results summary.

An email invitation to complete the online survey was sent to each CR program email via REDCap (SurveyMonkey in China), or by the Society leader to their distribution lists. The email requested that the “lead clinician” of each program complete the survey, and that program staff communicate to ensure only one survey be completed per program. Audit participation was voluntary, and electronic informed consent was obtained prior to survey initiation. Respondents were not asked to identify themselves or their programs to reduce the potential for socially desirable responding. Participants were offered free ICCPR CRFC online training [23], or to identify a member of their team to receive this free access. Data were collected between May 1^st^ and September 1^st^, 2025.

Up to two reminders were issued to non-responders at a two-week interval. ICCPR also distributed the survey invitation through their four social media channels, as well as their program and Council member email distribution lists. Audit leads and ICCPR Executive Board members also disseminated the survey invitation during CR-related conferences held in the data collection period. National leads were provided with their response percentage after one month, and where <40%, alternative strategies to enhance response were promoted, including providing a list of emails that failed delivery to identify an alternate, personal contact via phone or text, and distribution through Society social media channels or newsletters. Respondents with technical issues were provided the survey in PDF to complete, and responses were then entered by the Audit team to REDCap.

### Participants

The study population comprised all phase II CR programs identified worldwide. The study sample included all programs from the population that were successfully identified and contacted to participate in the Audit. Note that in the initial Audit only a random subsample of programs was contacted in countries with ≥250 programs.

The inclusion criterion was programs offering post-discharge services delivering at least: (1) an initial patient assessment; (2) structured aerobic exercise training, whether supervised or unsupervised (+/- technology); and (3) at least one additional core component [4] aimed at controlling cardiovascular risk factors (e.g., patient education, psychosocial or dietary counselling, tobacco cessation). Program staff were invited to contact the study email address where they were unsure about meeting these criteria, and some did.

Programs that operated solely and temporarily as part of a research study were excluded. Study materials made it explicit to potential participants that phase I or maintenance programs were not to be reported.

### Measures

The survey instrument was based on the 2016 Global CR Audit which comprised items harmonized across available national CR program surveys as described elsewhere [24], and some adaptations from the COVID-19 Rapid Audit survey [19]. The survey was updated for currency, brevity and clarity. The survey was publicly posted on the ICCPR website for three weeks to obtain input from the global CR community. It was revised accordingly. The full survey is available on ICCPR’s website (https://globalcardiacrehab.com/2025/26-Audit-Update).

Similar to the previous Audits, response options included numbers (constrained to plausible maximum and minimums to reduce human errors), closed-ended options as well as open-ended text (e.g., for “other” responses). Branching logic was applied to minimize respondent burden where applicable.

Initial items characterized respondent and program characteristics to describe the sample (e.g., year program opened). Country of program was coded into income classifications based on the World Bank [25]. Where available, comparable responses from the 2016 Audit [17,18,26] were extracted for comparison purposes, to enable inferential testing of decade change.

There was a section on centre-based models, as well as hybrid and alternative models / settings still meeting inclusion criteria. In centre-based CR, exercise sessions are primarily supervised by clinical staff and education is offered in-person [17]; patients are often directed to exercise unsupervised at home or in their community between sessions [4], and to review patient education materials independently [27]. In home-based models, exercise can be supervised using technology or unsupervised [10]. Education can be delivered individually with a patient by a member of the clinical team through technology (mobile phone, video call) or in a group [28]. As in traditional centre-based models, patients may also be encouraged to review educational materials independently [28]. Herein, hybrid CR is defined as subsuming what programs label ‘home-based’ CR, given delivery almost always requires at least one on-site visit to establish safety, but also for rapport [10,29]. Alternative CR models are defined as phase II CR delivered in community-based settings (maintenance programs excluded), or “other” settings (programs directed to specify open-ended for coding). Once the type of model was specified, survey items queried characteristics of any of these non-traditional models (i.e., hybrid [including home-based] and/or alternative [community-based or “other”-specified] settings) (Table 1).

**Table 1:** Cardiac Rehabilitation Model Operationalization.

**Phase**	**Model**	**Model Sub-Type**	**Description**
II programs offering post-discharge services delivering at least: (1) an initial patient assessment; (2) structured aerobic exercise training, whether supervised or unsupervised (+/- technology); and (3) at least one additional core component aimed at controlling cardiovascular risk factors (e.g., patient education, psychosocial or dietary counselling, tobacco cessation)	Centre-Based	n/a	Exercise sessions are primarily supervised by clinical staff and education is offered in-person. Patients are often directed to exercise unsupervised at home or in their community between sessions, and to review patient education materials independently.
	Alternative	Home-based	A structured program in which core CR components are delivered outside of a traditional centre, with patients monitored and supported remotely by healthcare professionals. Exercise can be supervised using technology or unsupervised. Education can be delivered individually with a patient by a member of the clinical team through technology (mobile phone, video call) or in a group. Patients may also be encouraged to review educational materials independently.
		Hybrid	Models combining centre- and home-based care. These models may be delivered with or without digital technologies, such as synchronous supervised group exercise classes via video streaming. Herein, hybrid CR subsumes what programs label ‘home-based’ CR, given delivery almost always requires at least one on-site visit to establish safety, but also for rapport
		Community-based	Comprehensive phase II CR delivered in community-based settings.

n/a, not applicable.

Survey items relevant to this sub-study included first whether alternative models were reimbursed (yes/no). With regard to availability, survey items queried whether hybrid and/or alternative CR models were offered (yes/no), and if yes, which types (i.e., home, community, and/or hybrid; if latter, program respondents were asked to specify details open-ended) and start year. Communication modes and their frequency of use were queried. Perceived barriers to communication with patients in these models (yes/no from a list of options based on the qualitative literature [30]) were also assessed. Program respondents were also asked what materials patients are provided to use to support them off-site (check all that apply). Programs were also asked to select all that apply from allocation criteria options, as well as acceptance of patient level of risk.

With regards to dose, programs were to report the number of times patients are asked to come on site; total sessions (i.e., product of total prescribed weeks x frequency / week but not including session length) was also computed separately for center-based and hybrid/alternative delivery models. With regards to volumes and capacity, items assessed the proportion of programs’ patients served in hybrid/alternative models annually (percentage, %), and perception of insufficient alternative model capacity. Finally, programs answered an open-ended item regarding strategies to increase hybrid/alternative CR model capacity.

### Data analysis

Data were analyzed using SPSS version 31 [31]. All initiated surveys were included in the analysis. Country information was missing for 18 (1.2%) initiated surveys; country response proportion was computed based on available responses.

Descriptive statistics, including frequencies and percentages for categorical variables, and medians with 25th–75th percentile ranges (P25-P75) for non-normally distributed continuous variables, were calculated using all available data, accounting for branching logic. Descriptive analyses regarding criteria to allocate patients to a hybrid/alternative model were complemented by a heat map illustrating the frequencies by country income class, created using Microsoft Excel. Open-ended responses were coded using content analysis [32]. Non-parametric tests were used where data were not normally distributed. Statistical significance was set at p < 0.05 throughout; however, where multiple comparisons were performed such as when comparing by income class, a more conservative threshold of p < 0.01 was applied. Where applicable, effect sizes for chi-square tests were quantified using Cramér’s V, and interpreted using conventional thresholds: 0.10 small, 0.30 moderate, and 0.50 large effects.

Inferential testing for differences by Audit assessment point (initial [18], COVID-19 [19] and current) were undertaken where appropriate, however caution is warranted in over-interpretation as these are repeated cross-sectional comparisons, with the COVID-19 sample skewed by pervasive program closures. Note it was not considered appropriate to test patient volume or capacity changes from the initial Audit given hybrid/alternative models were investigated more fulsomely in the Audit Update given changes since the pandemic.

Clustering of program-level data within countries was addressed using generalized linear mixed models (GLMM), specifying country as a random effect. Continuous outcomes (dose) were modeled assuming Poisson or negative binomial distributions, depending on dispersion. Residual distributions were examined to confirm model assumptions. Where significant, post-hoc pairwise Bonferroni comparisons were performed by income classes. Where a larger model was not warranted / exploratory purposes, as applicable chi-square, Kruskal–Wallis or Wilcoxon signed-rank tests were used as applicable.

## Results

### Audit Cohort

As detailed elsewhere [6], 113/195 (57.9%) countries in the world were confirmed to have any existent phase II CR programs (countries without CR shown in Fig 1 and Table 2, along with their Human Development Index [33] scores and annual ischemic heart disease (IHD) incidence (GBD 2023 [1], as a reflection of CR need); this is reported by country income class elsewhere (“Turk-Adawi K, Ghisi GLM, Arar N, et al. [Unpublished]”).

**Fig 1:**
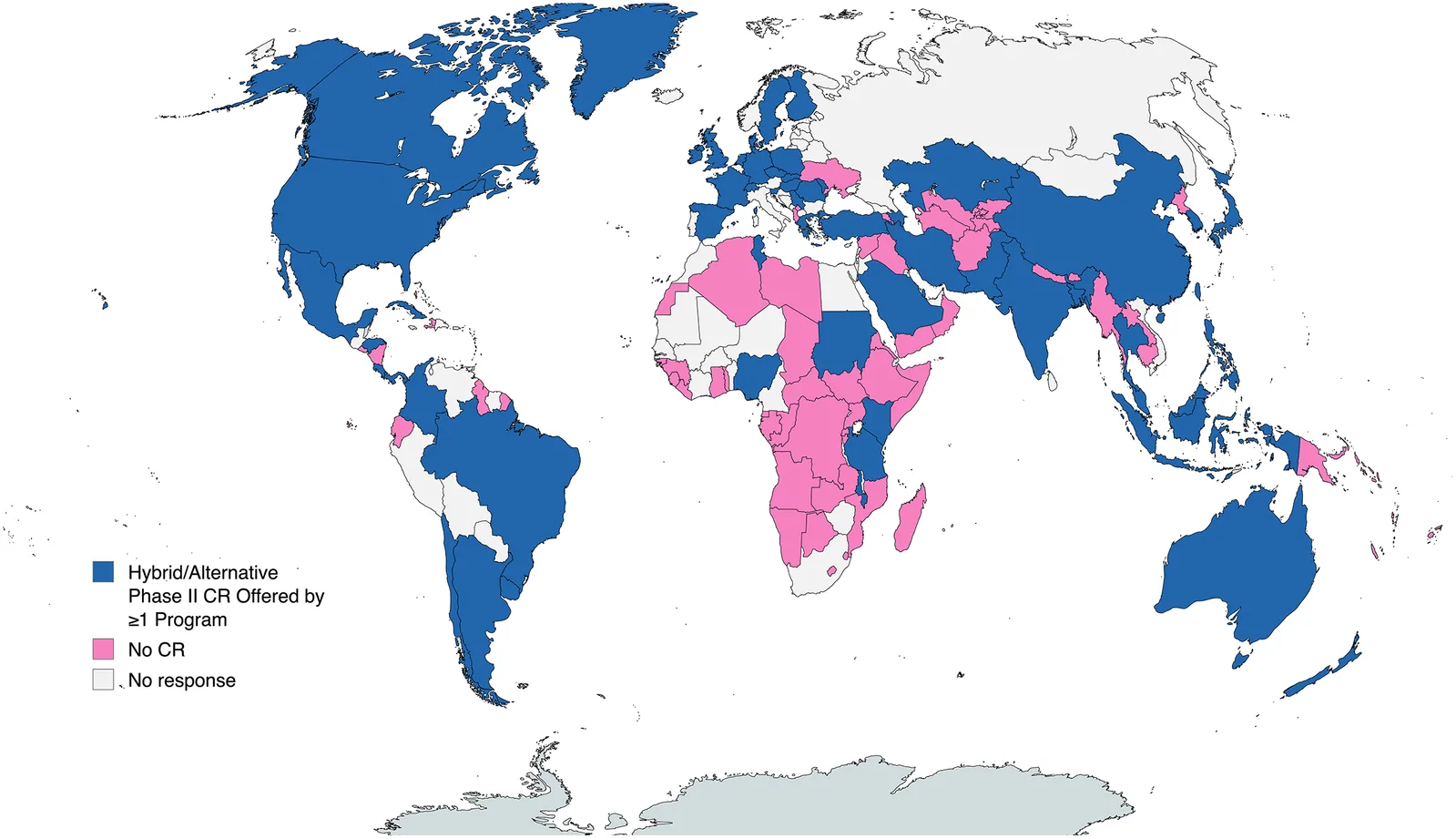
Global map depicting countries where hybrid/alternative phase II CR is reimbursed and where it is offered by ≥1 program. CR, cardiac rehabilitation. Note: No response denotes countries with confirmed CR availability where no program submitted a response that confirmed the availability of hybrid or alternative delivery models (n = 29 countries). Basemap obtained from Natural Earth (public domain): https://www.naturalearthdata.com/.

**Table 2:** Country-level contextual characteristics and cardiovascular burden^d^ as well as phase II cardiac rehabilitation volumes and capacity per program model by World Bank income classification [26].

Income class Country	Human Development Index ^c^	Annual IHD incidence 2023^a^	# CR programs in country	Median annual volumes per centre-based program	Median annual capacity per centre-based program	Median annual volumes per hybrid/alternative model program	Median annual capacity per hybrid/alternative model program
**Low-Income Countries**							
Afghanistan	0.496	34207	0	0	0	0	0
Burkina Faso	0.459	6796	1	NA	NA	NA	NA
Burundi	0.439	2854	0	0	0	0	0
Central African Republic	0.414	1900	0	0	0	0	0
Chad	0.416	4192	0	0	0	0	0
Cook Islands	NA	13	0	0	0	0	0
Democratic People’s Republic of Korea (North Korea)	NA	23213	0	0	0	0	0
Democratic Republic of Congo	0.522	24472	0	0	0	0	0
Eritrea	0.503	1943	0	0	0	0	0
Ethiopia	0.497	28903	0	0	0	0	0
Gambia	0.524	1012	0	0	0	0	0
Guinea-Bissau	0.514	633	0	0	0	0	0
Liberia	0.510	1858	0	0	0	0	0
Madagascar	0.487	7254	0	0	0	0	0
Malawi	0.517	3872	23	20	30	40	60
Mali	0.419	4623	1	NA	NA	NA	NA
Mozambique	0.493	9404	0	0	0	0	0
Niger	0.419	5180	1	NA	NA	NA	NA
Rwanda	0.578	3371	0	0	0	0	0
Sierra Leone	0.467	2500	0	0	0	0	0
Somalia	0.404	4249	0	0	0	0	0
South Sudan	0.388	1891	0	0	0	0	0
Sudan	0.511	80272	1	102	200	100	100
Syria	0.564	59540	0	0	0	0	0
Togo	0.571	3205	1	NA	NA	NA	NA
Uganda	0.582	8630	0	0	0	0	0
Yemen	0.470	45126	0	0	0	0	0
***LIC Total***	–	***371113***	***28***	***122***	***230***	***140***	***160***
***LIC Mean ± SD***	***0.487 ± 0.056***	***13745 ± 20076***	***1 ± 4***	***5 ± 21***	***10 ± 42***	***6 ± 22***	***7 ± 24***
***LIC Median (P25-P75)***	***0.496(0.439-0.517)***	***4249 (2222-16309)***	***0 (0−0)***	***0 (0−0)***	***0 (0−0)***	***0 (0−0)***	***0 (0−0)***
**Lower-Middle-Income Countries**							
Angola	0.616	9167	0	0	0	0	0
Bangladesh	0.685	198586	4	150	350	62	129
Benin	0.515	3831	2	NA	NA	NA	NA
Bhutan	0.698	926	1	NA	NA	NA	NA
Bolivia	0.733	6582	1	NA	NA	NA	NA
Cambodia	0.606	14683	0	0	0	0	0
Cameroon	0.588	8472	7	NA	NA	NA	NA
Comoros	0.603	455	0	0	0	0	0
Congo	0.649	1924	0	0	0	0	0
Cote d’Ivoire	0.582	9115	10	NA	NA	NA	NA
Djibouti	0.513	382	0	0	0	0	0
Egypt	0.754	253945	2	NA	NA	NA	NA
Eswatini (formerly Swaziland)	0.695	548	0	0	0	0	0
Federated States of Micronesia	0.615	501	0	0	0	0	0
Ghana	0.628	12717	0	0	0	0	0
Guinea	0.500	4420	0	0	0	0	0
Haiti	0.554	10087	0	0	0	0	0
Honduras	0.645	11800	3	NA	NA	500	600
India	0.685	2095969	80	40	120	15	50
Jordan	0.754	17262	1	NA	NA	NA	NA
Kenya	0.628	14490	4	150	500	137	215
Kiribati	0.644	74	0	0	0	0	0
Kyrgyzstan (Kyrgyz Republic)	0.720	13143	0	0	0	0	0
Laos	0.617	5392	0	0	0	0	0
Lebanon	0.752	11842	0	0	0	0	0
Lesotho	0.550	952	0	0	0	0	0
Mauritania	0.563	1894	3	NA	NA	NA	NA
Morocco	0.710	79006	4	NA	NA	NA	NA
Myanmar	0.609	50921	0	0	0	0	0
Nepal	0.622	39468	0	0	0	0	0
Nicaragua	0.706	6174	0	0	0	0	0
Nigeria	0.560	67369	3	30	100	12	15
Niue	NA	3	0	0	0	0	0
Pakistan	0.544	246505	8	170	600	8	60
Papua New Guinea	0.576	2344	0	0	0	0	0
Philippines	0.720	128394	25	225	300	20	45
Samoa	0.708	211	0	0	0	0	0
Sao Tome and Principe	0.637	91	0	0	0	0	0
Senegal	0.530	7581	8	85	175	NA	NA
Solomon Islands	0.584	555	0	0	0	0	0
Sri Lanka	0.776	35086	13	120	120	NA	NA
Tajikistan	0.691	17270	0	0	0	0	0
Tanzania	0.555	14581	2	321	2000	100	300
Timor-Leste	0.634	1038	0	0	0	0	0
Tunisia	0.746	20862	7	155	155	10	70
Uzbekistan	0.740	84528	0	0	0	0	0
Vanuatu	0.621	288	0	0	0	0	0
Vietnam	0.766	102177	3	20	90	NA	NA
Zambia	0.595	3097	0	0	0	0	0
Zimbabwe	0.598	8232	2	NA	NA	NA	NA
***LMIC Total***	–	***3624940***	***193***	***1466***	***4510***	***864***	***1484***
***LMIC Mean ± SD***	***0.639 ± 0.076***	***72499 ± 297888***	***4 ± 12***	***38 ± 76***	***116 ± 340***	***23 ± 86***	***40 ± 114***
***LMIC Median (P25-P75)***	***0.628 (0.584-0.706)***	***8794 (1252-19964)***	***0 (0-3)***	***0 (0-25)***	***0 (0-95)***	***0 (0−0)***	***0 (0−0)***
**Upper-Middle Income Countries**							
Albania	0.810	8829	0	0	0	0	0
Algeria	0.763	98998	0	0	0	0	0
Argentina	0.865	48552	24	50	60	5	5
Armenia	0.811	13384	0	0	0	0	0
Azerbaijan	0.789	33204	3	NA	NA	NA	NA
Belarus	0.824	62972	5	NA	NA	NA	NA
Belize	0.721	317	0	0	0	0	0
Bosnia and Herzegovina	0.804	11612	1	NA	NA	NA	NA
Botswana	0.731	1248	0	0	0	0	0
Brazil	0.786	327085	59	40	60	3	16
Cape Verde	0.668	420	0	0	0	0	0
China (not incl. Taiwan)	0.797	1906927	368	100	200	75	103
Colombia	0.788	68878	46	475	900	20	120
Cuba	0.762	28844	21	790	770	48	95
Dominica	0.761	118	1	NA	NA	NA	NA
Dominican Republic	0.776	14260	3	35	123	NA	NA
Ecuador	0.777	18345	0	0	0	0	0
El Salvador	0.678	7060	2	NA	NA	NA	NA
Equatorial Guinea	0.674	321	0	0	0	0	0
Fiji	0.731	926	0	0	0	0	0
Gabon	0.733	858	0	0	0	0	0
Georgia	0.844	32780	18	105	200	NA	NA
Grenada	0.791	192	1	NA	NA	NA	NA
Guatemala	0.662	12660	4	100	300	NA	NA
Indonesia	0.728	290834	17	100	300	NA	0
Iran	0.799	249062	38	110	275	NA	NA
Iraq	0.695	90365	0	0	0	0	0
Jamaica	0.720	2646	3	12	24	NA	NA
Kazakhstan	0.837	40826	10	500	350	5	100
Libya	0.721	17215	0	0	0	0	0
Malaysia	0.819	42385	9	810	725	50	500
Maldives	0.766	326	0	0	0	0	0
Marshall Islands	0.733	23	0	0	0	0	0
Mauritius	0.806	2088	0	0	0	0	0
Mexico	0.789	170254	65	50	120	5	40
Moldova	0.785	19563	1	NA	NA	200	200
Mongolia	0.747	4852	1	NA	NA	NA	NA
Montenegro	0.862	2368	1	NA	NA	NA	NA
Namibia	0.665	1326	0	0	0	0	0
North Macedonia	0.815	5893	1	NA	NA	NA	NA
Paraguay	0.756	8999	2	400	500	NA	NA
Peru	0.794	23523	12	100	250	NA	NA
Saint Lucia	0.748	160	0	0	0	0	0
Saint Vincent and Grenadines	0.798	188	0	0	0	0	0
Serbia	0.833	30602	5	550	110	300	300
South Africa	0.741	35207	23	50	200	NA	NA
Suriname	0.722	852	1	125	250	NA	NA
Thailand	0.798	80683	19	30	40	200	240
Tonga	0.769	78	0	0	0	0	0
Turkey	0.853	227450	13	130	175	32	35
Turkmenistan	0.764	13308	0	0	0	0	0
Tuvalu	0.689	10	0	0	0	0	0
Ukraine	0.779	324771	0	0	0	0	0
***UMIC Total***	–	***4384647***	***777***	***4662***	***5932***	***943***	***1754***
***UMIC Mean ± SD***	***0.767 ± 0.052***	***82729 ± 268913***	***15 ± 52***	***108 ± 208***	***138 ± 222***	***28 ± 69***	***50 ± 108***
***UMIC Median (P25-P75)***	***0.776 (0.731-0.799)***	***13308 (926-42385)***	***1 (0-10)***	***0 (0-100)***	***0 (0-200)***	***0 (0-5)***	***0 (0-38)***
**High-Income Countries**							
Andorra	0.913	163	0	0	0	0	0
Antigua and Barbuda	0.851	93	1	NA	NA	NA	NA
Australia	0.958	48122	396	200	240	21	30
Austria	0.930	30856	25	40	40	NA	NA
Bahamas	0.820	410	0	0	0	0	0
Bahrain	0.899	2564	2	80	3000	0	200
Barbados	0.811	464	1	NA	NA	NA	NA
Belgium	0.951	24069	60	400	200	NA	NA
Brunei Darussalam	0.837	411	2	30	20	NA	NA
Bulgaria	0.845	29862	1	NA	NA	NA	NA
Canada	0.939	72849	380	200	270	65	115
Chile	0.878	28806	46	80	112	27	40
Costa Rica	0.833	6063	11	100	146	500	500
Croatia	0.889	14774	3	300	200	NA	NA
Cyprus	0.913	1898	0	0	0	0	0
Czech Republic	0.915	39928	13	50	96	12	48
Denmark (including Greenland)	0.962	10275	98	800	800	10	10
Estonia	0.905	4188	2	NA	NA	NA	NA
Finland	0.948	18467	15	45	45	0	0
France	0.920	111077	144	388	450	15	35
Germany	0.959	362984	155	2000	1500	400	1000
Greece	0.908	33006	11	30	275	10	50
Guyana	0.776	588	0	0	0	0	0
Hungary	0.870	43132	23	40	150	NA	NA
Iceland	0.972	811	6	NA	NA	NA	NA
Ireland	0.949	10344	46	150	150	1	75
Israel	0.919	11470	17	550	358	75	120
Italy	0.915	310612	100	295	280	NA	NA
Japan	0.925	292531	678	55	97	1	8
Kuwait	0.852	11820	3	NA	NA	NA	NA
Latvia	0.889	9655	4	NA	NA	NA	NA
Lithuania	0.895	17528	193	1200	1200	NA	NA
Luxembourg	0.922	951	4	350	450	NA	NA
Malta	0.924	1245	1	500	500	NA	NA
Monaco	NA	80	0	0	0	0	0
Nauru	0.703	6	0	0	0	0	0
Netherlands & Aruba	0.955	31858	91	850	850	8	75
New Zealand	0.938	16546	34	75	120	20	100
Norway	0.970	10204	24	70	75	NA	NA
Oman	0.858	6201	4	NA	NA	NA	NA
Palau	0.786	24	0	0	0	0	0
Panama	0.839	4960	2	44	33	13	15
Poland	0.906	160869	60	300	400	50	150
Portugal	0.890	19070	28	125	160	NA	NA
Qatar	0.886	3547	1	88	150	44	20
Republic of Korea (South Korea)	0.937	51435	48	172	225	80	85
Romania	0.845	71245	23	8	75	50	200
Russia	0.832	817293	3	NA	NA	NA	NA
Saint Kitts and Nevis	0.840	59	0	0	0	0	0
San Marino	0.915	79	0	0	0	0	0
Saudi Arabia	0.900	49887	4	45	100	NA	NA
Seychelles	0.848	117	0	0	0	0	0
Singapore	0.946	15818	9	270	310	NA	NA
Slovakia (Slovak Republic)	0.880	20443	8	25	50	4	20
Slovenia	0.931	5614	10	100	145	12	50
Spain	0.918	65509	92	160	175	50	55
Sweden	0.959	28909	78	160	160	20	30
Switzerland	0.970	19380	72	120	150	4	10
Taiwan	NA	17100	52	75	120	NA	100
Trinidad and Tobago	0.807	2437	0	0	0	0	0
United Arab Emirates	0.940	13552	7	500	1000	NA	NA
United Kingdom	0.946	168399	223	402	450	100	198
Uruguay	0.862	4739	21	55	40	35	50
United States of America (& Guam & Puerto Rico)	0.938	1144557	2687	238	300	12	40
***HIC Total***	***–***	***4301953***	***6022***	***11765***	***15667***	***1639***	***3429***
***HIC Mean ± SD***	***0.896 ± 0.055***	***67218 ± 182786***	***94 ± 348***	***214 ± 343***	***285 ± 484***	***41 ± 99***	***84 ± 173***
***HIC Median (P25-P75)***	***0.911 (0.854-0.938)***	***14163 (2302 - 34737)***	***11 (2-54)***	***80 (30-283)***	***150 (40-290)***	***11 (0-37)***	***35 (0-85)***
**Not income classified** ^**b**^							
Venezuela	0.709	36611	5	115	105	NA	NA
**Global**							
***Total***	***–***	***12719264***	***7025***	***18130***	***26444***	***3586***	***6827***
***Mean ± SD***	***0.739 ± 0.151***	***65227 ± 229998***	***36 ± 204***	***113 ± 243***	***164 ± 358***	***27 ± 79***	***50 ± 126***
***Median (P25-P75)***	***0.762 (0.621-0.857)***	***10087 (1893-33706)***	***1 (0-11)***	***0 (0-110)***	***0 (0-175)***	***0 (0-12)***	***0 (0-49)***

NA, not available or missing data.

- not applicable.

CR, cardiac rehabilitation; HIC, high-income countries; IHD, ischemic heart disease; LIC, low income countries; LMIC, lower-middle income countries; SD, standard deviation; UMIC, upper-middle income countries.

^a^ Based on the Global Burden of Disease Study 2023 [1].

^b^ Based on World Bank [25].

^c^ https://hdr.undp.org/data-center/human-development-index#/indicies/HDI

^d^ Shaded columns: country-level Human Development Index and annual ischemic heart disease incidence estimates were included as contextual characteristics to facilitate interpretation of CR availability, volumes, and capacity relative to national development and the underlying burden of ischemic heart disease, respectively. More detail regarding incidence estimates in relation to CR need are found elsewhere (“Turk-Adawi K, Ghisi GLM, Arar N, et al. [Unpublished]”).

Overall, ≥ 1 survey was initiated by programs in 90/113 countries (79.6% country response proportion; n = 23/1505, 1.5% had no country specified; countries without responses by income class is also reported elsewhere [6] (“Turk-Adawi K, Ghisi GLM, Arar N, et al. [Unpublished]”)).

Overall, 6,905 CR programs were identified globally (“Turk-Adawi K, Grace SL, Arar N, et al. [Unpublished]”); number of programs by country is reported elsewhere (“Turk-Adawi K, Ghisi GLM, Arar N, et al. [Unpublished]”) [20]. In total, *1,505* programs initiated a survey. The median program response proportion across countries was 62.3% (P25-P75 = 24.1–100.0%); response proportions by country income classification are reported elsewhere (“Turk-Adawi K, Ghisi GLM, Arar N, et al. [Unpublished]”). In the initial Audit, country (111 countries, 84% response) and program response proportions (32%, with only a random subsample of centers in countries ≥250 programs) globally were consistent [17].

### Reimbursement of hybrid/alternative models

Overall, 286/1233 (23.2%) programs reported hybrid/alternative models as reimbursable by government or insurance companies in their region (shown by country in Fig 1 and Table 3). These programs were situated across 40 countries (35.4% of 113 countries with any available CR, or 20.5% of 195 countries internationally).

**Table 3:** Hybrid / alternative model availability, reimbursement by government or insurance companies and proportion served in these models, by country with CR and income class.

Income Class^a^ Country with CR	Hybrid/alternative models reimbursed, n (%) N = 1,505	Programs reporting availability of hybrid/alternative models, n (%) N = 1,505	Proportion (%) of program’s new patients served in hybrid/alternative model per year^a^ (n = 371)
**Low**	**4.2% (2.1-6.2)**	**4.2% (2.1-6.2)**	**35.0% (20.0-35.0)**
Malawi	1 (8.3%)	1 (8.3%)	40.0 (40.0-40.0)
Sudan	SPP	SPP	SPP
**Lower-Middle**	**0.0% (0.0-3.7)**	**38.8% (25.0-44.1)**	**10.0% (5.0-40.0)**
Bangladesh	SCS	SCS	SCS
Cameroon	SPP	SPP	SPP
Côte D’Ivoire	0 (0.0%)	3 (100.0%)	–
Honduras	SCS	SCS	SCS
India	4 (7.4%)	24 (42.1%)	15.0 (5.0-30.0)
Jordan	SPP	SPP	SPP
Kenya	0 (0.0%)	2 (50.0%)	137.0 (60.0-137.0)
Mauritania	SPP	SPP	SPP
Nigeria	SCS	SCS	SCS
Pakistan	2 (25.0%)	3 (37.5%)	8.0 (6.0-8.0)
Philippines	0 (0.0%)	4 (40.0%)	19.5 (4.5-81.0)
Senegal	0 (0.0%)	0 (0.0%)	–
Sri Lanka	0 (0.0%)	0 (0.0%)	–
Tanzania	SCS	SCS	SCS
Tunisia	0 (0.0%)	1 (33.3%)	10.0 (10.0-10.0)
Vietnam	SCS	SCS	SCS
Zimbabwe	SPP	SPP	SPP
**Upper-Middle**	**9.6% (0.0-22.3)**	**23.7% (9.3-38.0)**	**20.0% (5.0-50.0)**
Argentina	1 (10.0%)	1 (10.0%)	5.0 (5.0-5.0)
Azerbaijan	SPP	SPP	SPP
Brazil	11 (28.9%)	15 (39.5%)	3.0 (0.0-10.0)
China	82 (36.3%)	107 (47.3%)	75.0 (45.0-200.0)
Colombia	5 (14.3%)	1 (2.9%)	20.0 (20.0-20.0)
Cuba	SCS	SCS	SCS
Dominican Republic	SCS	SCS	SCS
Georgia	0 (0.0%)	0 (0.0%)	–
Guatemala	SCS	SCS	SCS
Indonesia	1 (9.1%)	1 (9.1%)	–
Iran	1 (25.0%)	1 (25.0%)	–
Jamaica	SPP	SPP	SPP
Kazakhstan	0 (0.0%)	1 (11.1%)	5.0 (5.0-5.0)
Malaysia	0 (0.0%)	3 (60.0%)	50.0 (30.0-50.0)
Mexico	8 (13.8%)	13 (22.4%)	5.0 (0.5-9.0)
Moldova	SPP	SPP	SPP
Montenegro	SPP	SPP	SPP
Paraguay	SPP	SPP	SPP
Peru	1 (8.3%)	0 (0.0%)	–
Serbia	0 (0.0%)	2 (50.0%)	300.0 (100.0-300.0)
South Africa	SPP	SPP	SPP
Suriname	SPP	SPP	SPP
Thailand	3 (33.3%)	3 (30.0%)	200.0 (100.0-200.0)
Turkey	0 (0.0%)	2 (33.3%)	31.5 (13.0-31.5)
**High**	**20.0% (9.5-28.1)**	**17.4% (5.6-30.4)**	**20.0% (7.5-50.0)**
Australia	46 (54.1%)	43 (50.0%)	21.0 (4.5-61.3)
Austria	SCS	SCS	SCS
Bahrain	SPP	SPP	SPP
Barbados	SPP	SPP	SPP
Belgium	SCS	SCS	SCS
Brunei Darussalam	SCS	SCS	SCS
Canada	14 (35.0%)	23 (54.8%)	65.0 (10.0-200.0)
Chile	6 (25.0%)	4 (16.0%)	27.0 (7.5-458.0)
Costa Rica	3 (37.5%)	1 (12.5%)	500.0 (500.0-500.0)
Croatia	SPP	SPP	SPP
Czech Republic	0 (0.0%)	1 (11.1%)	12.0 (12.0-12.0)
Denmark (incl. Greenland)	SCS	SCS	SCS
Finland	2 (50.0%)	1 (25.0%)	–
France	5 (26.3%)	6 (31.6%)	15.0 (0.0-51.3)
Germany	1 (20.0%)	1 (20.0%)	400.0 (400.0-400.0)
Greece	2 (33.3%)	3 (50.0%)	10.0 (5.0-10.0)
Hungary	0 (0.0%)	1 (20.0%)	–
Ireland	6 (17.6%)	8 (23.5%)	–
Israel (& Palestine)	SCS	SCS	SCS
Italy	0 (0.0%)	0 (0.0%)	–
Japan	4 (11.1%)	1 (2.6%)	–
Lithuania	SPP	SPP	SPP
Luxembourg	SPP	SPP	SPP
Malta	SPP	SPP	SPP
Netherlands (& Aruba)	8 (9.3%)	5 (5.8%)	8.0 (5.3-40.0)
New Zealand	7 (58.3%)	6 (50.0%)	20.0 (10.0-61.0)
Norway	1 (11.1%)	0 (0.0%)	–
Panama	SCS	SCS	SCS
Poland	4 (20.0%)	6 (30.0%)	50.0 (0.0-50.0)
Portugal	0 (0.0%)	0 (0.0%)	–
Qatar	SPP	SPP	SPP
Romania	SCS	SCS	SCS
Saudi Arabia	SCS	SCS	SCS
Singapore	1 (25.0%)	0 (0.0%)	–
Slovak Republic	SPP	SPP	SPP
Slovenia	0 (0.0%)	1 (20.0%)	12.0 (12.0-12.0)
South Korea	3 (13.6%)	3 (13.0%)	80.0 (80.0-80.0)
Spain	8 (17.0%)	9 (18.8%)	50.0 (42.5-50.0)
Sweden	3 (20.0%)	1 (5.9%)	20.0 (20.0-20.0)
Switzerland	SPP	SPP	SPP
Taiwan	2 (9.5%)	1 (4.8%)	–
United Arab Emirates	0 (0.0%)	0 (0.0%)	–
United Kingdom^b^	17 (53.1%)	29 (87.9%)	100.0 (50.0-339.0)
United States of America	13 (26.0%)	6 (11.3%)	12.0 (8.5-72.5)
Uruguay	1 (20.0%)	2 (40.0%)	35.0 (20.0-35.0)
**Global** ^**c**^	**286 (23.2%)** ^**d**^	**371 (29.5%)** ^**e**^	**20.0 (5.0–50.0)** ^**f**^

- indicates missing data (no response received).

^a^ median (P25-P75) shown.

^b^ includes also Bermuda.

^c^ program surveys with country not income unclassified and country unknown (n = 23) included in global row only to optimize generalizability.

Note: n (valid %) shown.

CR, cardiac rehabilitation

SPP: result Suppressed to protect Program Privacy (i.e., only 1 program in country).

SCS: result suppressed because estimates are unreliable due to Small Cell Size (≤3 programs responding).

^d^ Differences in reimbursement of hybrid/alternative cardiac rehabilitation delivery models by income class were assessed using chi-square tests (χ²(4)=24.60, p < 0.001; Cramér’s V = 0.141). Analyses used item-specific denominators.

^e^Differences in the availability of hybrid/alternative cardiac rehabilitation delivery models by country income classification were assessed using chi-square tests (χ²(4)=12.31, p = 0.015; Cramér’s V = 0.099). Analyses used item-specific denominators.

^f^Differences in the number of new patients served annually through hybrid/alternative models by income class were assessed using the Kruskal–Wallis test (H(3)=10.86, p = 0.012).

When asked to specify which elements / models were reimbursed, globally, most commonly this was: community-based programs (n = 114/1505, 7.6%), asynchronous exercise or education as part of an overall program (n = 109, 7.2%), group synchronous exercise sessions (video; n = 84, 5.6%), 1−1 exercise sessions with clinician (video; n = 81, 5.4%), 1−1 non-exercise appointment(s) with a clinician (audio or video; n = 70, 4.7%), group synchronous education session(s) (video; n = 70, 4.7%), and ‘other’ (n = 22, 1.5%). This is shown by income class in Fig 2. “Other” unique responses were named proprietary or branded CR models (e.g., Heart Manual, Cardihab), mobile applications (i.e., “Apps”), and provision of home monitoring equipment (e.g., electrocardiogram, blood pressure).

**Fig 2:**
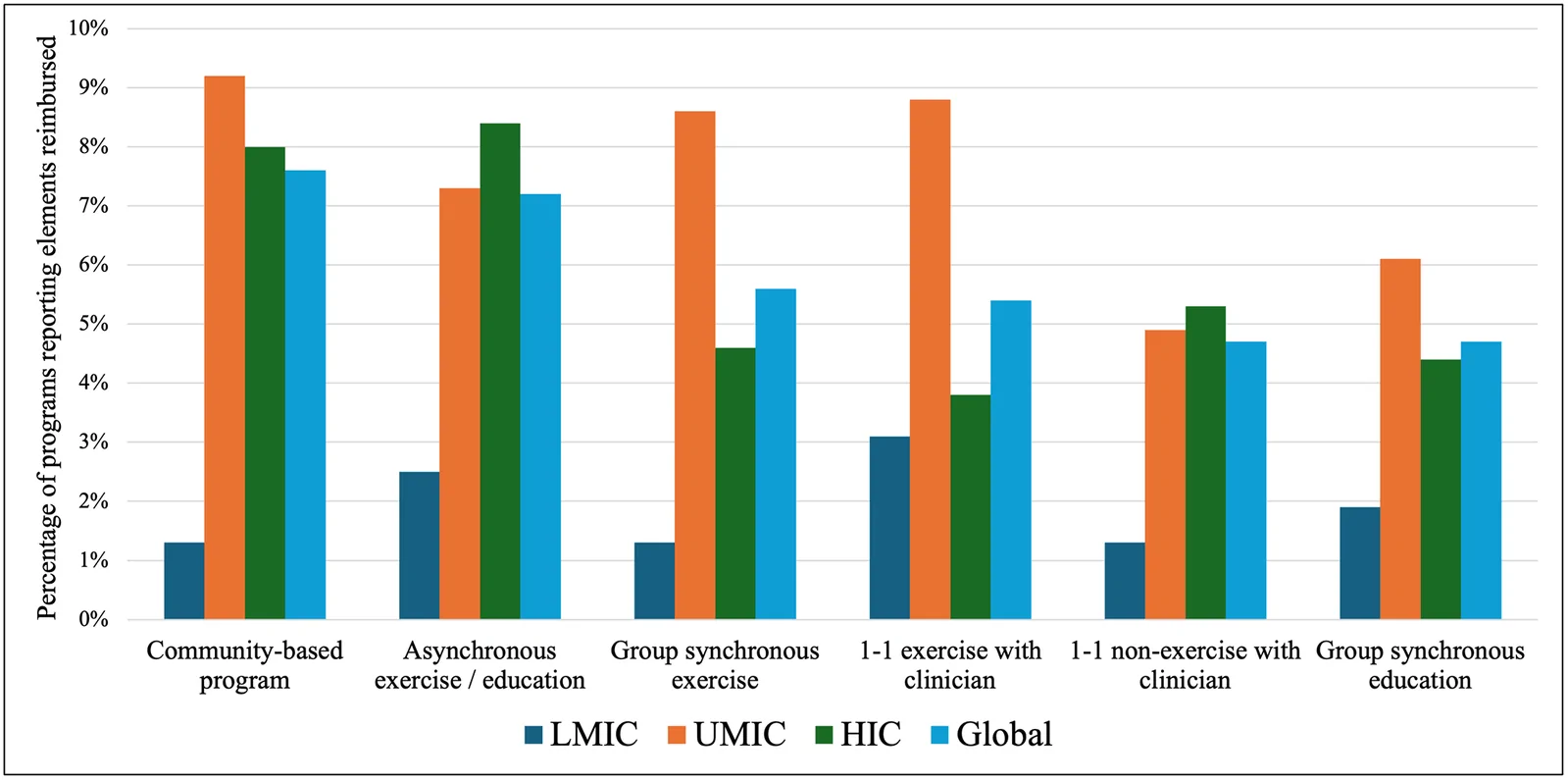
Alternative program elements/models reimbursable by government or insurance by country income class^a,b^. Note: high-income countries (HIC), upper-middle-income countries (UMIC), and lower-middle-income countries (LMIC), as defined by the World Bank [25]. ^a^ Programs selected all elements that apply; valid percent (%) shown. ^b^ Among all responses (N = 1505). Global estimates include low-income and non-income-classified/unknown countries; low-income countries are not shown separately due to small numbers (n = 2).

By income class, 163/286 (57.0%) programs in high-income countries (HICs), 114 (39.9%) in upper-middle income countries (UMICs), 7 (2.4%) in lower-middle income countries (LMICs), and 1 (0.3%) in a low-income country (LIC) reported reimbursement of hybrid/alternative models by government or insurance providers; one additional program (0.3%) was located in a non-income classified country (i.e., Venezuela; Table 3). This represented statistically significant variation across classes (χ²(4)=24.60, p < 0.001), of small-to-moderate effect (Cramér’s V = 0.141).

In the 2016 Initial Audit [18], 130 (14.5%) programs reported these models as reimbursed (χ²(1)=24.79, p < 0.001 vs. 2025). In the Rapid COVID-19 Audit in 2020 [19], 169 (21.0%) programs reported these models as reimbursed (χ²(1)=1.36, p = 0.24 vs. 2025).

As reported elsewhere [6], respondents often reported multiple program funding sources across models. Among programs reporting that hybrid/alternative models were reimbursed, 130/286 (45.5%) reported that patients still paid part or all of the cost of CR out-of-pocket; where patients paid, these programs reported they paid a median of 35.0% of the total program cost (P25-P75 = 20.0–80.0%). Among programs responding that hybrid/alternative models were not reimbursed, 458/947 (48.4%) reported that patients paid part or all of the cost of CR out-of-pocket (median = 60.0% of the total program cost [P25-P75 = 30.0–100.0%]; p < 0.001).

### Global availability and structure of hybrid/alternative models

Globally, 371/1258 (29.5%) programs offered some form of hybrid/alternative model (Fig 1 and Table 3 by country), with 269/371 (72.5%) of those home-based, 151 (40.7%) offering hybrid, and 141 (38.0%) community-based services. Content analysis of open-ended responses revealed substantial heterogeneity in program descriptions of their hybrid delivery models. Most programs described requiring an initial in-person assessment prior to transition to home-based delivery (64%). Step-down models – whereby patients completed an initial period of centre-based CR followed by supervised home-based rehabilitation – were the next most-commonly reported (56%). Nearly half of programs (49%) incorporated on-going supervision of home-based exercise through telephone or tele-health follow-up. Technology-enabled delivery – including videoconferencing platforms, Apps, and remote physiological monitoring – was reported by 41% of programs. Next, 30% of programs described flexible or individualized hybrid pathways tailored uniquely to meet patient needs. Integration with community-based settings (e.g., gyms, local physiotherapy services) was reported by 23% of programs. A smaller proportion (19%) reported minimally-supervised or low-intensity hybrid models, typically involving limited in-person contact with remote follow-up.

Finally, few programs reported ‘other alternative’ phase II delivery models open-ended. These included digitally-integrated treatment pathways (33.3%), prescription-based or de-centralized specialist delivery models without clinic attendance (33.3%), and CR delivered through primary care or payer-linked management plans, such as health fund or general practitioner-led telephone programs (33.3%).

In their hybrid/alternative model, globally, 67/333 (20.1%) of programs reported not having any prescribed on-site visits for patients (i.e., fully home-based); other programs offering this model request patients come on-site a median of 2 times/program (P25-P75 = 1–4). Median on-site visit requirements were significantly higher in UMICs (median = 3; P25-P75: 1–4; Kruskal–Wallis H(3)=25.90, p < 0.001).

Relatedly, 160/344 (46.5%) programs ‘always’ and 115 (33.4%) ‘mostly’ (i.e., exceptions made on case-by-case basis) request that patients come on-site for a post-program assessment. The requirement for patients to attend an on-site post-program assessment differed significantly by country income classification (χ²(8)=28.29, p < 0.001; Cramér’s V = 0.20). Specifically, programs in UMICs were more likely to report consistently requiring on-site post-program assessments, whereas programs in HICs more frequently reported flexibility, including partial or fully remote assessments. Among programs requesting on-site post-program assessments, they estimated a median of 70.0% (P25-P75 = 40–90) of patients oblige; this too differed significantly by country income classification (Kruskal–Wallis H(2) = 23.01, p < 0.001), with higher attendance reported by programs in HICs (median = 90.0%) compared with those in UMICs (median = 70.0%) and LMICs (median = 65.0%).

Of the programs offering any hybrid/alternative CR models, 165/363 (45.5%) programs reported these models as reimbursable by government or insurance companies in their region (χ²(6)=25.07, p < 0.001, vs 54.5% programs not offering these models). At least some programs (not necessarily all given healthcare policy often varies by region within countries) in 48 countries offering hybrid/alternative models reported these services as not reimbursed, Countries spanned all World Bank income groups [25], with the largest numbers of programs reporting non-reimbursement located in China, India, Australia, United Kingdom, Canada, Brazil, and Mexico.

Availability of hybrid/alternative CR delivery models also differed significantly by country income class (χ²(4)=12.31, p = 0.015), although the magnitude of association was small (Cramér’s V = 0.099; Table 3). Post-hoc pairwise χ² tests with Bonferroni correction (α = 0.01) indicated that availability was significantly higher in UMICs compared with HICs (p = 0.006). Differences between LMICs and HICs (p = 0.05) and between LMICs and UMICs (p = 0.80) did not reach the corrected significance threshold.

In the 2016 Initial Audit [18], **285 (31.1%)** programs reported offering hybrid and/or alternative models of CR, a proportion that did not differ from that reported in the 2025 Global Audit (χ²(1)=0.65, p = 0.42). **Among programs offering alternative models, 82 (30.5%)** reported these models as reimbursable in 2016 [18]. As reported in the Rapid COVID-19 Audit [19], 150 (18.5%) programs reported offering hybrid and/or alternative CR models immediately before the COVID-19 pandemic was declared, with 172 (21.2%) programs offering such models during the pandemic. This was **significantly lower than the proportion reported in the 2025 Global Audit** (χ²(1)=17.10, p < 0.001), which could reflect that most programs were at least temporarily closed in 2020 [19]. **Among programs offering alternative models, 131 (41.5%)** reported these models as reimbursable during the pandemic.

In this 2025 Audit Update, programs globally reported that these hybrid/alternative models were initiated early in the pandemic (median year = 2020; P25-75: 2018–2023). By income class (Kruskal–Wallis H(3)=10.05, p = 0.018), the median year of model initiation was significantly more recent in UMICs and LICs (median = 2021).

Finally, program-reported differences in the nature of centre-based and hybrid/alternative models are shown in Table 4, including by income class. One-fifth of programs reported consistency between the two models for patients or that patient care is individualized based on need so it depends, but a further one-fifth of programs reported patients do not receive as comprehensive care. Less than one-fifth of programs reported that patients get access to fewer disciplines, but over 10% reported that patients get more 1−1 staff time (albeit less total time with staff per program). “Other” differences reported were few: variation in staff contact intensity based on logistical factors or payment structure, and limitations in delivering certain assessments virtually despite access to a multidisciplinary team.

**Table 4:** Any differences in program characteristics and staffing between centre-based and hybrid/alternative cardiac rehabilitation models (where available), by World Bank country income classification and globally.

	Income Class			Global^a^ N=371
	HICs (n=173)	UMICs (n=154)	LMICs (n=40)	
It depends on the patient’s needs, and if they want to come on site for some specific components.	43 (24.9%)	34 (22.1%)	10 (25.0%)	87 (23.5%)
We do not do as comprehensive an assessment for the alternative model, so they are likely not getting the same care as patients in the centre-based model.	16 (9.2%)	59 (38.3%)	3 (7.5%)	79 (21.3%)
Patients in hybrid/alternative model do not receive as many core components.	23 (13.3%)	42 (27.3%)	9 (22.5%)	74 (19.9%)
Patients in the hybrid/alternative program model do not meet with the same variety of healthcare professionals as patients in the centre-based model (e.g., they have a case manager who handles care / liaises as needed).	25 (14.5%)	30 (19.5%)	9 (22.5%)	65 (17.5%)
Patients in the hybrid/alternative program get more 1-1 staff time, but less total time with staff than patients in the centre-based model.	24 (13.9%)	15 (9.7%)	5 (12.5%)	44 (11.9%)
Patients in the hybrid/alternative program are advantaged in some ways in terms of comprehensiveness and access to specialists compared to patients in the centre-based model.	4 (2.3%)	8 (5.2%)	3 (7.5%)	15 (4.0%)
Other	7 (4.0%)	0 (0.0%)	0 (0.0%)	7 (1.9%)
It is hybrid, so patients get the same.	29 (16.8%)	41 (26.6%)	9 (22.5%)	80 (21.6%)
Patients get the same comprehensiveness of care and multidisciplinary team access in both models.	32 (18.5%)	37 (24.0%)	8 (20.0%)	77 (20.8%)

Note: frequency and valid % shown.

HIC, high-income countries; UMIC, upper-middle income countries; LMIC, lower-middle income countries, based on World Bank [25].

^a^ Data for low-income countries are not shown due to the small number of responses (n = 2). Includes two additional programs respondents from a non-income classified or unknown country.

### Implementation of hybrid/alternative models: Staffing, communication and patient resources

In hybrid/alternative models, the following healthcare provider types interact with patients: nurses (n = 195/371, 52.6%), physiotherapists (n = 183, 49.3%), physicians (n = 140, 37.7%), exercise physiologists or kinesiologists (n = 133, 35.8%) (note in some countries, the exercise training component is most often delivered by physiotherapists, while in others it is delivered by various types of exercise specialists) [3]. In programs reporting physicians directly interacting with patients in hybrid/alternative models, the most common specialty was general cardiology (n = 31), and cardiologists with subspecialty training in preventive cardiology or CR specifically (n = 10) were also reported. Next were physical medicine and rehabilitation (physiatrists; n = 13), sports medicine (n = 3) and primary care physicians (n = 2). Other non-physician provider types interacting with patients in these models were: dietitians/nutritionists (n = 14), psychologists (n = 7), and occupational therapists (n = 4). Less commonly reported were social workers, technicians, and therapy/healthcare assistants (each n = 2). Single mentions were: other allied health provider (unspecified), care navigator, pharmacist, and one program specified no direct staff-patient interaction. Any interaction of the main clinician types with patients is shown by income class in Fig 3. Across income classes, any type of exercise professional were the most consistently reported providers interacting directly with patients in hybrid/alternative CR models, particularly in LMICs. In contrast, physician involvement varied substantially by income class, being lowest in HICs, and highest of any profession in UMICs.

**Fig 3:**
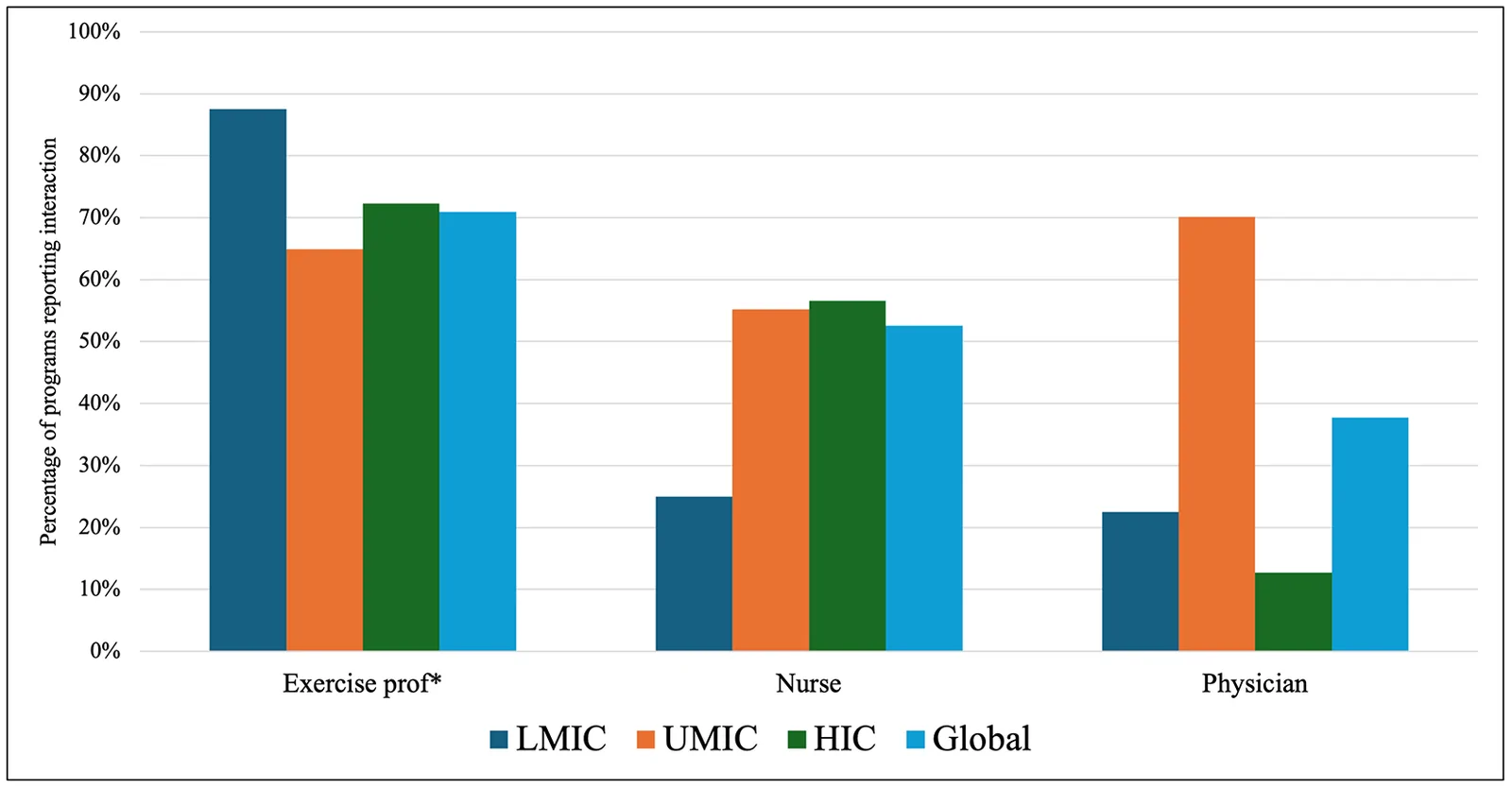
Healthcare provider types interacting with hybrid/alternative model patients by country income class^a^. Prof, professionals. *included physiotherapists, kinesiologists, and exercise specialists. Valid percent (%) shown; respondents were directed to check all that apply to these pre-specified response options. HIC, high-income countries; UMIC, upper-middle income countries; LMIC, lower-middle income countries; based on World Bank [25]. ^a^ Among programs reporting availability of hybrid/alternative CR models (N = 371). Data for low-income countries are not shown due to the small number of responses (n = 2). Two additional programs responded from a non-income classified or unknown country (not included in figure).

Healthcare provider types and numbers interacting with patients in centre-based models are reported elsewhere, including by country income class and Audit decade [3]. The most common provider types on CR teams (full or part-time) were: **nurses** (n = 816, 88.1%), **dieticians** (n = 739, 80.2%), **physiotherapists** (n = 733, 79.3%; plus n = 477, 52.2% other exercise professionals), and cardiologists (n = 721, 77.9%) [3].

Forms of communication between patients and program staff along with their frequency of use are depicted in Fig 4. As shown, some of the more commonly-used modes globally such as smartphone Apps, email, and internet/webpage-based communication were “never” used in some programs, with some variation by income class (e.g., email, text, telephone landline). Other forms of communication reported were few: postal correspondence, use of specific social media-based messaging platforms (i.e., WeChat), and transmission of physiologic data via electrocardiographic (ECG) telemonitoring.

**Fig 4:**
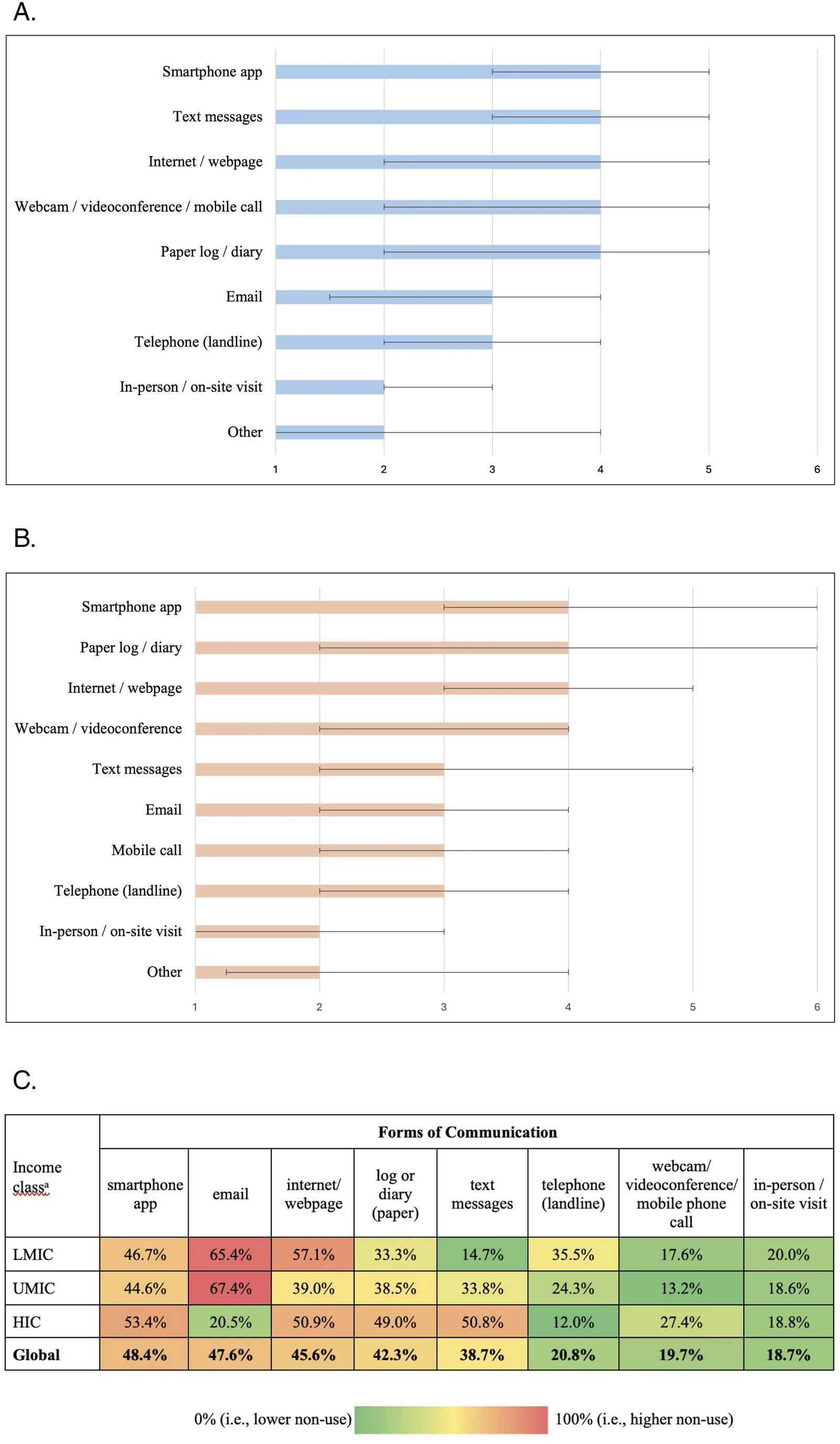
Frequency* of patient communication by mode in hybrid/alternative delivery models in phase II cardiac rehabilitation programs globally. **A. Most Frequently Used Forms in 2025.** Note: median frequency depicted, with whiskers showing P25-P75; *frequency response options were coded as follows: ‘just once’ = 1; ‘monthly’ = 2; ‘several times / month’ = 3; ‘weekly’ = 4; ‘several times per week’ = 5; and ‘daily’ = 6. **B. Most Frequently Used Forms in 2016 [18].** Note: median frequency depicted, with whiskers showing P25-P75; frequency response options were coded as follows: ‘just once’ = 1, ‘monthly’ = 2, ‘several times / month’ = 3, ‘weekly’ = 4; ‘several times per week’ = 5, and ‘daily’ = 6. Note 2: landline and mobile phone were assessed as separate forms of communication in the 2016 Audit survey. **C. Forms of patient communication never used by programs in hybrid/alternative delivery models where offered, by country income classification in 2025.** Valid percent (%) shown. HIC, high-income countries; UMIC, upper-middle income countries; LMIC, lower-middle income countries; LIC, low-income countries, based on World Bank [25]. ^a^ Data for low-income countries are not shown due to the small number of responses (n = 2). Two additional programs were reported from a non-income classified or unknown country (not included in figure).

Frequency of use by communication modality in the initial Audit is also depicted in Fig 4 for comparison [18]; while not inferentially tested given some measurement changes, median frequency rankings were largely consistent across 2016 and 2025 Audits, with digital modalities predominating. Text messaging increased in frequency in the 2025 Audit, while in-person visits remained least frequent in both audit years.

Globally, 196/334 (58.7%) programs perceived barriers to communication with patients in the hybrid/alternative model; of these programs, the specific barriers most-commonly encountered were: patients lacking technology access or proficiency, following by internet connectivity issues for patients or staff (Supplemental Figure 1); the latter appeared slightly more commonly-reported in LMICs.

Other barriers for these models reported in 2025 were (Supplemental Figure 1): staff time constraints (n = 2), patient motivation or adherence challenges unrelated to technology (n = 2), mismatch between patient communication platform preferences and available program tools (n = 1), privacy or regulatory restrictions on communication modalities (n = 1), and insufficient program funding or resources to support communication infrastructure (n = 1).

By time, in the 2016 Initial Audit [18], 61 (49.6%) programs perceived such barriers to communication. As reported in the Rapid COVID-19 Audit [19], 80 (63.0%) programs perceived barriers to communication with patients in the hybrid/alternative model before COVID-19, and 102 (62.6%) perceived barriers during COVID-19.

Program leads were also asked to report what materials patients were provided, or expected to use, to support achievement of CR goals outside of the main clinical centre. While 39/371 (10.5%) programs reported that no materials were provided, most programs reported supplying educational materials (n = 252, 67.9%), activity trackers (either provided directly or through the use of patients’ personal mobile phone applications; n = 140, 37.7%), resistance training equipment such as therapeutic elastic bands (n = 120, 32.3%), and other materials (n = 38, 10.2%). Among these, the only unique items reported were aerobic exercise equipment, most commonly cycle ergometers (n = 6).

Provision of materials did not differ significantly by country income classification, whereas reporting that no materials were provided did, with programs in HICs (n = 7/175, 4.0%) less likely to report providing no such materials than other programs in other income classes (χ²(4)=24.25, *p* < 0.001). In the 2016 Audit [18], educational materials were also the most-commonly provided support resource in hybrid/alternative model programs globally (n = 114, 68.7%), followed by activity trackers (n = 58, 34.9%), with resistance training materials reported infrequently (n = 27, 16.3%) [18].

### Dose by model

Full details regarding centre-based CR dose are reported elsewhere [3]. For hybrid/alternative models (Table 5), programs prescribed a median of 10 sessions (P25–P75 = 4–24) internationally, comprising a median of 1 session per week (P25–P75 = 0.5–2.5) over 8 weeks (P25–P75 = 6–12; note session length was not assessed but in centre-based was one hour quite invariably [3]). Although program duration did not differ significantly between hybrid/alternative and centre-based models (Wilcoxon signed-rank test: Z=−1.76, p = 0.08), in hybrid/alternative models patients were prescribed significantly fewer sessions per week (Z=−7.09, p < 0.001). Consequently, the total number of prescribed CR sessions was significantly lower in hybrid/alternative models compared with centre-based models (Z=−5.62, p < 0.001).

**Table 5:** Sessions Prescribed (Dose) per phase II Cardiac Rehabilitation Program by World Bank Income Classification, Model and Audit Decade.

Country Income Class^a^	CR Model							
	Centre-Based^b^				Hybrid/Alternative Setting^c^			
	Program duration (weeks)	Frequency (sessions/ week)	Total Sessions 2025 (/pt./program)	Total Sessions 2016 [27] (/pt./program)	Program duration (weeks)	Frequency (sessions/ week)^d^	Total Sessions 2025 (/pt./program)	Total Sessions 2016 [27] (/pt./program)
HICs	10.0 (7.0-14.0)	2.0 (2.0-3.0)	23.0 (12.0-28.0)	20.0 (12.0-30.0)	8.0 (8.0-12.0)	1.0 (0.5-2.2)	11.0 (6.0-24.0)	6.0 (3.0-16.0)
UMICs	8.0 (5.0-12.0)	3.0 (2.0-3.0)	24.0 (12.0-36.0)	33.5 (16.0-44.3)	8.0 (4.0-12.0)	1.0 (0.5-2.5)	9.0 (3.0-24.0)	11.0 (5.6-39.5)
LMICs	6.0 (4.0-12.0)	3.0 (3.0-3.0)	18.0 (12.0-30.0)	12.0 (10.5-24.0)	6.0 (4.0-12.0)	1.6 (0.7-3.0)	12.0 (3.0-18.0)	9.0 (6.0-9.0)
**Global** ^**e**^	**8.0 (6.0-12.0)**	**2.0 (2.0-3.0)**	24.0 (12.0-36.0)*	24.0 (12.0-36.0)**	**8.0 (6.0-12.0)**	**1.0 (0.5-2.5)**	**10.0 (4.0-24.0)**†	**6.2 (3.0-17.5)**††

Abbreviations: pt., patient. Acronyms: CR, cardiac rehabilitation; HIC, high-income countries; UMIC, upper-middle income countries; LMIC, lower-middle income countries.

Note: median (P25-P75) shown.

- missing data.

^a^ based on World Bank [25]. Data for low-income countries are not shown due to the small number of responses (n = 2). Includes two additional programs respondents from a non-income classified or unknown country.

^b^ includes hospitals or other clinical settings.

^c^ includes home-based (+/- technology), hybrid, community-based phase II.

^d^ formal contact with CR staff, which may be via phone or other technology.

^e^ includes surveys of unknown or unclassified countries.

*p < 0.001 for difference by country income class, tested using a generalized linear mixed model. Bonferroni-adjusted post-hoc tests indicated that programs in UMICs prescribed significantly more sessions than those in HICs (*p* < 0.001) and LMICs (*p* = 0.002).

**p = 0.07 for difference by Audit decade, tested using a generalized linear mixed model.

†p = 0.94 for difference by country income class, tested using a generalized linear mixed model.

††p = 0.17 for difference by Audit decade, tested using a generalized linear mixed model.

As also shown in Table 5, the total number of prescribed CR sessions for hybrid/alternative models did not differ significantly by country income class (F(4,311)=0.20, p = 0.94); substantial variability within income groups was observed. Similarly, no significant difference was observed between the 2016 and 2025 Audits (F(1,418)=1.91, p = 0.17).

### Model allocation criteria, annual patient volumes and capacity

Globally, programs most commonly reported offering hybrid/alternative models on the basis of: patient choice, distance to centre, transportation barriers, risk stratification, patient indication, time or work constraints during CR centre hours, and cost. Several programs reported another basis, namely: health system or service availability constraints, such as absence of local rehabilitation centres or home-based delivery as the only available option (n = 3), clinical characteristics not explicitly captured by risk stratification, including post-operative status, advanced age, or severe deconditioning (n = 2), and program-level resource limitations (n = 1).

A heat map in Fig 5 depicts allocation criteria by income classification. In the initial 2016 Audit [18], programs reported basing model allocation on these criteria at the following frequencies: patient choice (n = 121/166, 72.9%), transportation barriers (n = 110, 66.3%), distance to centre (n = 108, 65.1%), time or work constraints during CR centre hours (n = 84, 50.6%), risk stratification (n = 76, 45.8%), patient indication (n = 67, 40.4%), and cost (n = 38, 22.9%).

**Fig 5:**
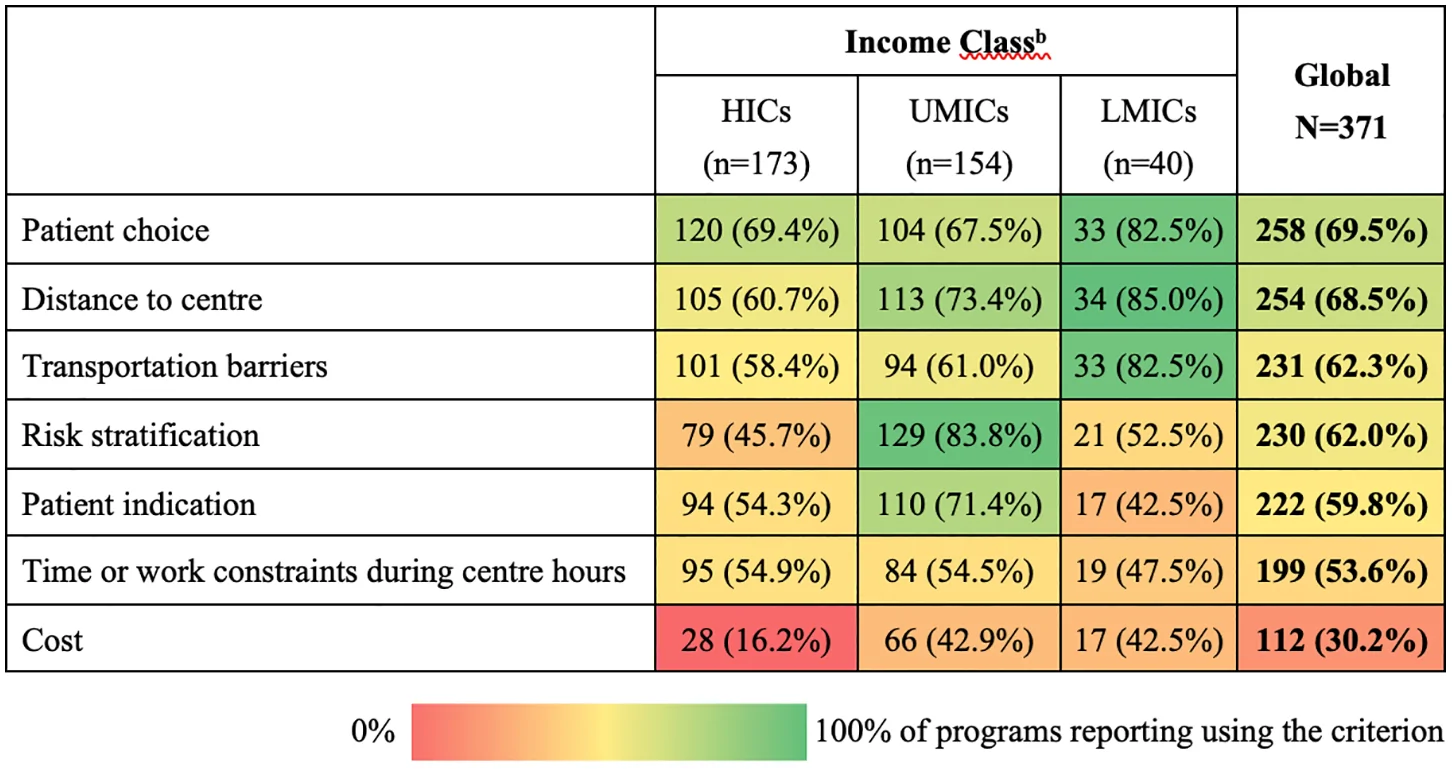
Model Allocation Criteria^a^ where Hybrid/Alternative Settings Offered, by Country Income Class. Note: n (valid percent; %) programs shown. CR, cardiac rehabilitation. ^a^ respondents selected all that apply. ^b^ HIC, high-income countries; UMIC, upper-middle income countries; LMIC, lower-middle income countries, based on World Bank [25]. Data for low-income countries are not shown due to the small number of responses (n = 2). Two additional programs responded from a non-income classified or unknown country (not included in figure).

Overall, 202/371 (54.4%) programs accept moderate-risk patients in their hybrid/alternative model, with 80 (21.6%) accepting high-risk patients (almost 80% of programs accepted low-risk patients, and only 5% indicated they do not risk stratify). Acceptance of high (χ²(4)=5.14, p = 0.27) and moderate-risk (χ²(4)=3.86, p = 0.43) patients did not differ by country income classification. In the 2016 Initial Audit [18], 88 (68.8%; χ²(1)=8.74, p = 0.003, Cramér’s V = 0.13; vs 2025) programs accepted moderate-risk patients in their hybrid/alternative model, with 35 (27.3%) accepting high-risk (χ²(1)=2.24, p = 0.13 vs 2025).

Globally, programs offering hybrid/alternative models treated one in five of their annual patients in these models (Table 3). This proportion differed significantly by income classification (Kruskal–Wallis H(3)=10.86, p = 0.012; Table 3), with lower proportions of hybrid patients observed in LMICs; estimates for LICs should be interpreted cautiously due to small sample size. By time, this was 10.0% (P25-75: 5−26) in the 2016 Initial Audit (Z = −2.13, p = 0.03 vs 2025) [18] – and as per the Rapid COVID-19 Audit – 5.0% (P25-75: 0−25) immediately before COVID-19 (Z = −7.17, p < 0.001 vs 2025) and 90.0% (P25-75: 34−100) during COVID-19 (Z = −9.91, p < 0.001 vs 2025) [19].

Annual hybrid/alternative CR model patient volumes and capacity are juxtaposed with traditional centre-based volumes and capacity by country in Table 2 (capacity considered in more detail elsewhere (“Turk-Adawi K, Ghisi GLM, Arar N, et al. [Unpublished]”). Globally, programs served a median volume of **50** new patients per year in hybrid/alternative models (P25-P75: **10−100**), with a reported annual capacity of **70** patients (P25–P75: **30−200**). Program-reported annual patient volume and annual capacity in hybrid/alternative models were strongly positively correlated (Spearman’s rho = 0.733, p < 0.001). In comparison, programs reported higher annual centre-based patient volumes (median = **135**, P25–P75: **5−400;** Z = −10.06, p < 0.001) and capacity (median = **200**, P25–P75: **88−500**; Z = −9.19, p < 0.001).

Annual patient volumes and capacity in hybrid/alternative CR models are also shown by country income classification in Table 2. While LICs were excluded from inferential analyses due to the small number of responding programs (n = 2), volumes differed significantly (H(2) = 10.54, p = 0.005), with post-hoc pairwise comparisons with Bonferroni adjustment revealing that programs in UMICs served a significantly higher volume of patients annually than those in HICs (Z = −2.59, p = 0.010) and LMICs (Z = −2.62, p = 0.009); no other difference was observed. With regard to annual patient capacity, this too differed significantly by income class (H(2)=10.88, p = 0.004); post-hoc analyses again indicated that programs in UMICs reported significantly greater capacity than those in HICs (Z = −2.85, p = 0.004) and LMICs (Z = −2.45, p = 0.014).

Globally, 160/350 (45.8%) programs perceived they did not have sufficient capacity in their hybrid/alternative model to meet demand; by income class this was: 57 (36.8%) in HICs, 86 (56.6%) in UMICs, 15 (38.5%) in LMICs, and 2 (100.0%) in LICs (χ²(4)=16.26, p = 0.003; Cramér’s V = 0.22; again caution is warranted in interpreting the LIC value). Reasons most commonly were: not enough staff (n = 130/160, 81.3%), not being fully funded (n = 73, 45.6%), patients’ level of risk too high for unsupervised exercise (n = 71, 44.4%), patients lacking the technological/other capability (n = 52, 32.5%), and patients do not request it (n = 47, 29.4%). A few programs reported another reason: limitations in infrastructure, equipment, or technological systems (n = 4), patient-level contextual barriers, including language barriers or limited family engagement (n = 2), program efficiency and workflow constraints that favored group-based delivery over individualized care (n = 1), lack of sufficient cost-effectiveness evidence (n = 1), lack of insurance coverage for these models (n = 1), and post-pandemic reductions in operational capacity, whereby models implemented during COVID-19 were no longer feasible (n = 1).

In the 2016 Initial Audit [18], 54 (38.0%) programs perceived they did not have sufficient capacity in their hybrid/alternative model to meet demand (χ²(1)=2.51, p = 0.11 vs 2025). The first three reasons outlined above were also assessed in that earlier survey; at that time, 42 (25.3%) programs selected not enough staff, 31 (18.7%) programs selected not being fully funded, and 6 (5.2%) selected patients’ level of risk was too high. As reported in the Rapid COVID-19 Audit [19], 147 (50.9%) programs perceived they did not have sufficient capacity in their hybrid/alternative model to meet demand before COVID-19 (χ²(2)=6.36, p = 0.04 vs 2025), with 121 (42.2%) programs perceiving insufficient capacity during COVID-19 (χ²(4)=21.64, Cramer’s V = 0.12, p < 0.001 vs 2025).

Finally, 298/371 (80.3%) programs offering these models described at least one enabling factor to significantly increase their program’s hybrid/alternative model capacity. As shown in Supplemental Figure 2, content analysis revealed these were most commonly increasing staffing and human resources, improving digital infrastructure, funding/cost reduction, and increasing education/awareness.

## Discussion

This global Audit Update provides the most current and comprehensive examination to date of hybrid and alternative CR models worldwide. As evidence has accumulated suggesting comparable efficacy of home-based and alternative CR models relative to traditional centre-based delivery [12], there have been increasing calls to expand their availability as a strategy to address the persistent under-capacity of CR globally [34, 35]. Leveraging ICCPR’s network, this Audit is unique in its global scope, high country- and program-level response proportions, and its ability to characterize contemporary practice following the rapid expansion of alternative delivery models during the COVID-19 pandemic. Overall, approximately one-quarter of programs reported reimbursement for hybrid/alternative models, available in roughly one-third of countries with CR globally. Notably, despite increased evidence supporting the effectiveness of hybrid and alternative CR models and their widespread uptake during the COVID-19 pandemic [19,36], their overall availability does not appear to have substantially increased compared with the initial 2016 ICCPR Audit [18]. This suggests that pandemic-related expansion was largely temporary and not translated into sustained integration of alternative models within routine CR delivery. Encouragingly, programs delivering these models reported multidisciplinary patient care and provision of education, exercise tracking and resistance training materials to patients to support their secondary prevention. However, compared with centre-based models, in hybrid/alternative programs, patients were generally prescribed a lower dose of CR, and in one-fifth of programs patients received fewer core components. While patient choice and clinical factors appropriately guided CR model allocation, patient cost emerged as an increasingly prominent deterrent. UMICs appeared to be leading implementation efforts, yet substantial barriers related to staffing, financing, and digital access persist.

### Reimbursement and availability of hybrid and alternative models

Reimbursement for hybrid and alternative CR models remains limited. Fewer than one-quarter of programs reported that these models were reimbursed, stemming from only approximately one-third of countries where CR exists. While given the nature of the design no causal conclusions can be drawn, a clear gradient was observed by country income classification, as is observed for universal health coverage more broadly [37], with reimbursement negligible in LICs and LMICs. Even where reimbursement was reported through government or insurance mechanisms, patients appeared to continue to bear a substantial proportion of delivery costs, with reported averages of approximately one-third of total program costs, although this was notably lower than the out-of-pocket burden observed where programs were not reimbursed. Reimbursement was more frequently reported where these models were available, underscoring that reimbursement, while necessary, is insufficient on its own to eliminate financial barriers to participation (but again, causal conclusions cannot be drawn.

Among programs reporting reimbursement, community-based models and asynchronous exercise or education sessions were most commonly covered, particularly in HICs, whereas one-to-one or group education sessions were more frequently reimbursed in MICs. Notably, reimbursement for hybrid and alternative models was reported to be higher during the pandemic [19] and, in some cases, higher in the initial ICCPR audit than at present [18], suggesting a retrenchment of financial support as emergency measures expired.

Availability of hybrid and alternative CR models varied in parallel with reimbursement patterns. Approximately 30% of programs reported offering such models, most commonly home-based, although over one-third of these were delivered in community settings. Programs with reimbursement were substantially more likely to offer hybrid or alternative models. While given the cross-sectional design, this association cannot establish whether reimbursement increases availability, it does suggest policy advocacy might be a potential lever to expand funding as a mechanism to increase availability. Interestingly, availability was reported as more common in LMICs than in UMICs, despite lower reimbursement, suggesting that some programs may be innovating out of necessity. Availability rates were similar to that reported in 2016 [18], but appeared lower during the COVID period [19], potentially reflecting widespread program closures at that time.

Importantly, the proportion of programs offering hybrid or alternative models was similar to that reported in 2016 [18] and, in some settings, lower than during the pandemic period [19], although a higher proportion of a program’s patients were allocated to these models than in 2016 [18]. While caution in interpretation is warranted given the repeated cross-sectional design, this suggests that gains in availability achieved during COVID-19 were not maintained; this potentially reflects a withdrawal of temporary reimbursement mechanisms and emergency policy flexibilities, not necessarily a lack of feasibility or acceptability of these models.

### Nature, implementation, and dose of hybrid and alternative models

In terms of program structure and implementation, approximately one-fifth of programs reported that the overall content of hybrid or alternative models was equivalent to centre-based CR, and a similar proportion indicated that these models enabled greater individual tailoring to patient needs. However, concerns regarding comprehensiveness were also evident: around 20% of programs reported less comprehensive assessments and consequently fewer risk factors addressed, particularly in UMICs, and one-fifth reported delivery of fewer core CR components, although this was less common in HICs.

The dose of CR delivered through hybrid and alternative models was consistently lower than that of centre-based programs, with a median of 10 sessions, typically delivered once weekly over approximately eight weeks. This dose was observed across country income classifications and remained stable relative to prior ICCPR Audits [18,19]. While 20% of programs reported no on-site visits, those incorporating in-person contact delivered a median of two visits per patient. Post-program assessments were most-commonly conducted on-site, reported by approximately 80% of programs, with higher rates in UMICs; however, programs perceived patient greater attendance at on-site assessments in HICs, suggesting ongoing access barriers elsewhere.

Patients participating in hybrid and alternative models were exposed to multidisciplinary teams, most frequently including exercise professionals, nurses, and physicians. Physician involvement was reported more often in UMICs, potentially reflecting differences in workforce availability, remuneration structures, or scope of practice. Communication modalities revealed a bimodal pattern: programs tended to rely either on high-technology solutions (e.g., Apps, web platforms, email) or low-technology approaches. Email communication was less common in MICs, while text messaging was more frequently used and landline telephone use was less prevalent in LMICs. Compared with 2016 [18], texting was more commonly used, reflecting broader global shifts in communication practices.

### Model allocation criteria

Allocation of patients to hybrid or alternative CR models was primarily patient-driven, with approximately 70% of programs citing patient choice as a key criterion, reflecting patient-centredness [38]. Importantly, clinical considerations also featured prominently: patient risk level and clinical indication were each reported by roughly 60% of programs, and were more frequently cited in UMICs. This suggests that as hybrid and alternative models expand in these settings, implementation is increasingly aligned with contemporary evidence and clinical guidance. Compared with the 2016 Audit [18], clinical factors were more heavily weighted, representing a positive shift toward evidence-informed practice.

Distance and transportation barriers were also commonly cited allocation criteria, consistent with patient-reported determinants of model selection in prior research [14]. These considerations were particularly salient in lower-income settings, where patient resource constraints are greater. Conversely, cost was identified as a factor influencing allocation in approximately 30% of programs, representing an increase from the initial Audit [19]. This finding is concerning; financial barriers continue to deter participation even where alternative models are available. Acceptance of high-risk patients into hybrid or alternative models was reported by approximately 20% of programs, and remained consistent across income groups and over time [16,17], although acceptance of moderate-risk patients appeared lower than in 2016, a finding that may warrant further investigation.

### Patient volumes and barriers to scale-up

Despite their potential to address CR under-capacity [39], hybrid and alternative models currently serve a modest proportion of patients. Where available, approximately one-fifth of a program’s annual CR patients were enrolled in these models, with lower proportions observed in lower-income settings. Programs reported serving a median of 50 patients annually through hybrid or alternative delivery, indicating limited scale. Volumes and capacity were higher in UMICs and were reported to be greater during the COVID period [19], likely reflecting both necessity-driven adoption and temporary policy supports [40].

Approximately half of programs reported barriers to expanding hybrid or alternative CR delivery, a proportion similar to that reported in 2016 [18] but lower than during the pandemic [19]. The most-commonly cited barriers were insufficient staffing, inadequate financial support, and limited digital assets, with these challenges reported more frequently in low-income countries and UMICs. These findings highlight persistent structural constraints that limit the scalability of alternative CR models, even where feasibility and acceptability have been demonstrated.

### Implications for practice, policy, and research

From a practice perspective, findings suggest that while hybrid and alternative CR models are being delivered by a multidisciplinary team and with patient-centred allocation, attention is needed to ensure adequate dose and comprehensive coverage of core CR components. Programs may benefit from guidance on minimum standards for content and dose that preserve effectiveness while maintaining flexibility, such as is offered in the models module of ICCPR’s CR Foundations Certification online training for multidisciplinary professionals [23].

Policy implications are substantial. Reimbursement was strongly associated with availability, yet remains limited and, in some cases, lower than during the pandemic. Policy-makers should consider sustained funding mechanisms for hybrid and other alternative CR models as one potential strategy to expand secondary prevention services and reduce inequities in access. The ICCPR and affiliated professional Societies are well positioned to advocate for harmonized policy approaches and to support member countries in integrating alternative models into health systems [41,42]. The lack of sustained growth in hybrid and alternative CR availability represents a missed opportunity. The pandemic demonstrated that rapid scale-up is feasible when policy, reimbursement, and organizational supports align. Failure to maintain these gains risks perpetuating global CR under-capacity despite the availability of effective delivery models. Consideration of dose should be foremost in implementation planning.

Research priorities include evaluating strategies to increase program scale without compromising quality, understanding patient outcomes associated with lower-dose delivery, and identifying cost-effective implementation approaches across diverse settings. Further work is also needed to address digital inequities and to develop low-tech solutions that maintain fidelity to evidence-based CR.

### Limitations

This Audit has several limitations. Chiefly, non-response bias is possible**.** Although the recruitment approach was designed to achieve broad international representation, responding programs may differ systematically from non-responding programs in characteristics relevant to this sub-study, including program resources, reimbursement, delivery models, and use of digital technologies. If programs with greater resources or more established services were more likely to respond, the availability of hybrid/alternative models, reimbursement, and digital technology use may be over-estimated. Conversely, programs with fewer resources or limited capacity may have been less likely to respond, particularly in lower-resource settings. Therefore, the findings should be interpreted as describing responding programs rather than providing definitive estimates of global hybrid/alternative CR availability or reimbursement.

Relatedly, while global in scope, representativeness may be limited, particularly in LICs, where programs were under-represented. In addition, within-country heterogeneity may not be fully captured; in large countries especially, CR penetration and practices may vary substantially across regions. Therefore, data reported by participating programs may not fully reflect national-level availability or service delivery.

Data were self-reported and as such may be subject to social desirability bias, with additional potential for variability due to health system, cultural, and language differences across regions. Comparisons over time are constrained by the repeated cross-sectional design, particularly related to program closures during the COVID period.

With regard to analyses, there are considerations related to country-level clustering and statistical inference. Programs were nested within countries, and country-level factors such as reimbursement mechanisms, healthcare systems, and regulatory frameworks may have influenced program characteristics. The number of responding programs also varied substantially across countries, meaning that countries with larger numbers of programs contributed more observations to program-level analyses. For analyses where appropriate, country was included as a random effect in GLMM to account for clustering; however, not all descriptive analyses or comparisons were amenable to multi-level modeling. Consequently, some observations may not have been statistically independent, and estimates from program-level analyses should be interpreted with consideration of potential country-level clustering and unequal country representation. Finally, multiple inferential comparisons increase the risk of type I error; findings should be interpreted cautiously.

## Conclusions

In this global Audit Update, hybrid and alternative CR models were available in approximately one-third of programs and reimbursed in only one-quarter, despite growing evidence supporting their effectiveness and their potential to address global CR under-capacity. While models were generally delivered with multidisciplinary involvement and patient-centred allocation, they were characterized by lower dose and, in some cases, reduced comprehensiveness compared with centre-based CR. Financial, staffing, and digital barriers appear to constrain scale-up, with notable disparities by country income classification. These findings highlight the potential need for sustained reimbursement, implementation support, and policy alignment to realize the full potential of hybrid and alternative CR models in advancing equitable global cardiovascular care.

## Supporting information

S1 Fig Program-Perceived Barriers to Communication with Patients in Hybrid/Alternative Cardiac Rehabilitation Models^a^ by World Bank Country Income Classification^b^. ^a^Valid percent (%) shown; respondents were directed to check all that apply to these 5 pre-specified response options. HIC, high-income countries; UMIC, upper-middle income countries; LMIC, lower-middle income countries, based on World Bank [25]. ^b^Among programs reporting availability of hybrid/alternative CR models (N = 371). Data for low-income countries are not shown due to the small number of responses (n = 2). Two additional programs responded from a non-income classified or unknown country (not included in figure).
(TIFF)

S2 Fig Program-Recommended Strategies to increase Hybrid / Alternative Cardiac Rehabilitation Program Model Capacity^a^. BP, blood pressure, HR, heart rate, ECG, electrocardiogram. ^a^ n = 298 open-ended responses content analyzed [32]. Totals exceed 100% because some respondents reported more than one.
(TIFF)
